# Structure‐reactivity relationships between the fluorescent chromophores and antioxidant activity of grain and sweet sorghum seeds

**DOI:** 10.1002/fsn3.350

**Published:** 2016-02-16

**Authors:** Minori Uchimiya, Xinzhi Ni, Ming Li Wang

**Affiliations:** ^1^ USDA‐ARS Southern Regional Research Center1100 Robert E. Lee BoulevardNew OrleansLouisiana70124; ^2^ USDA‐ARS Crop Genetics and Breeding Research Unit2747 Davis RoadTiftonGeorgia31793; ^3^ USDA‐ARS Plant Genetic Resources Conservation UnitGriffinGeorgia30223

**Keywords:** Cereal, colorimetric method, proanthocyanidin, sweet sorghum, transition metal

## Abstract

Polyphenolic structures are the putative cause of a variety of seed functions including bird/insect resistance and antioxidant activity. Structure‐reactivity relationships are necessary to understand the influence of polyphenolic chromophore structures on the tannin content and free radical quenching ability determined by the traditional calorimetric methods. This study investigated the relationships between the structural attributes of fluorescent chromophore and the following seed characterization methods: procyanidin (by acid‐butanol assay) and flavonoid (by vanillin assay) contents, radical quenching (by DPPH assay), electron‐donating capacity (by Fe^III^ reduction), and *λ*_max_ (by UV/visible spectrophotometry). Distinctively different response was observed for different seed categories: U.S. grain sorghum hybrids, African grain sorghum, and sweet sorghum. The U.S. grain sorghum varieties (low‐tannin to maximize the livestock digestion) responded only to the DPPH assay. For sweet sorghum and African grain sorghum, linear correlation was observed between (1) the antioxidant activity (2) the amounts of procyanidins and flavonoids, and (2) the aromaticity of fingerprint fluorescent structures.

## Introduction

Based on the hypothesis that phenol‐containing structures (including procyanidin, flavonoids, and short oligomers of phenolic structures) are responsible for the bird/insect resistance of sorghum seeds, a number of standardized tannin methods have been established (Butler 1982). For example, 4,8 linkages of procyanidin are cleaved in the acid‐butanol assay, while vanillin assay targets meta‐substituted flavonoids present in the terminal units of procyanidin (Butler 1982). Modified tannin methods have been developed to estimate the degree of polymerization based on the ratio from vanillin and acid‐butanol assays (Butler 1982). However, these standardized tannin methods are influenced by the experimental artifacts, including the solvent composition (Xu and Chang 2007), kinetically controlled reactions, overlapping spectra near the detection wavelength, low sensitivity, and the reactivity of nontarget structures (Butler 1982). Structure‐reactivity relationships are necessary to understand the influence of polyphenolic chromophore structures on the tannin content and free radical quenching ability determined by the traditional calorimetric methods.

The objective of this study was to investigate the structure‐reactivity relationships between fluorescent chromophores and procyanidin (by acid‐butanol assay) and flavonoid (by vanillin assay) contents, radical quenching (by DPPH assay), electron‐donating capacity (by Fe^III^ reduction), and *λ*_max_ (by UV/visible spectrophotometry). Fourteen U.S. grain sorghum hybrids, four African grain sorghum, and four sweet sorghum samples were selected, in order to investigate diverse seed categories.

## Materials and Methods

Distilled, deionized water (DDW) with a resistivity of 18 MΩ cm (APS Water Services, Van Nuys, CA) was used in all procedures. All chemical reagents were obtained from Sigma‐Aldrich (Milwaukee, WI) with the highest purity available.

### Sorghum seed samples

African grain sorghum seeds (*Sorghum bicolor* accessions) were received at National Plant Germplasm System (NPGS) (USDA, 2014) from east and central Africa in 1960s, and are hereby denoted (parenthesis provides NPGS accession identifier): Seed 1 (PI 276776), Seed 2 (PI 282857), Seed 3 (PI 329552), and Seed 4 (PI 267650). Table S1 section provides physical characteristics of each seed (GENESYS, 2015). Four sweet sorghum accessions were obtained from NPGS and are hereby denoted by the variety: Dale (PI 651495, brown colored), Theis (PI 651497, light brown), M81E (PI 653411, dark brown), and Keller (PI 653617, brown). Fourteen U.S. grain sorghum hybrids (hereby denoted 44XX) were obtained from different commercial sources: Athens Seed, Lawn and Garden, Watkinsville, GA (4401 and 4432); DeKalb, St. Louis, MO (4422); Dyna‐Gro, Richmond, CA (4433); Gayland Ward, Hereford, TX (4419); Sorghum Partners, New Deal, TX (4402, 4434, 4411, 4425, 4437, 4435, 4409); and Southern States, Richmond, VA (4426, 4436). As the word of caution, the U.S. grain sorghum hybrids were highly unstable, and molded within 2 week of storage at 25°C, and were infested by the storage insects thereafter. All seeds were analyzed immediately upon receipt, before the visible molding and insect infestation occurred. The total of 22 seed samples (African grain, U.S. grain, and sweet sorghum) were extracted in triplicate using methanol and acetone/water (70/30 v/v) by end‐over‐end rotation (70 rpm) at 20 g L^−1^ for 72 h. Extracts were filtered (0.45 *μ*m Millipore Millex‐GS; Millipore, Billerica, MA) and analyzed immediately. All analyses were performed using the triplicate extracts of each seed sample.

### Ferrozine, acid‐butanol, vanillin, DPPH, and total metal contents of seed samples

Electron‐donating capacity of each seed sample was investigated by (1) reduction of Fe^III^ by acetone‐water (70:30 v/v) extracts under acidic pH and (2) quantification of Fe^II^ product using ferrozine colorimetric assay (Stookey 1970). Acidic pH was utilized to (1) make Fe^III^ reduction by dihydroxybenzene thermodynamically favorable (see E_h_‐pH diagram in Fig. S1) (Uchimiya and Stone 2006), (2) minimize oxidation of Fe^II^ product by O_2_ (Kanzaki and Murakami 2013), and (3) minimize Fe^II^ complexation by phenolic components of extracts (Martell et al. 2004). Acetone‐water extract (0.2 mL) and FeCl_3_ stock solution (1 mmol L^−1^ in 0.5 mol L^−1^ HCl) were added to DDW to yield 50 *μ*mol L^−1^ FeCl_3_ in 5 mL total volume. Reactors were allowed to stand for 30 min, and then 0.2 mL of resulting solution was added to 5 mL ferrozine stock solution (3‐(2‐Pyridyl)‐5,6‐diphenyl‐1,2,4‐triazine‐p,p’‐disulfonic acid monosodium salt hydrate; 0.1 g L^−1^ in 0.5 mol L^−1^ MOPS buffer at pH 7) (Stookey 1970). Absorbance at 562 nm (Stookey 1970) was determined using diode‐array UV/visible spectrophotometer (HP8452A, Hewlett‐Packard, Palo Alto, CA) with DDW as the blank immediately. Five‐point calibration was obtained using 1.0 mol L^−1^ FeCl_2_ stock solution prepared in 1.0 mmol L^−1^ HCl daily to minimize autoxidation. Control experiments indicated negligible Fe^II^ loss by complexation and autoxidation.

Total Fe, K, Mg, P, Ca, Mn, Na, and Zn concentrations of selected seed samples (seed 3, seed 4, 4401, and 4437) were determined by duplicate extraction of 1.25 g seed in 25 mL of 0.2 mol L^−1^ ammonium oxalate (pH 3.5) by end‐over‐end rotation (70 rpm) for 24 h in the dark. Filtered (0.45 *μ*m) extracts were acidified to 4 vol% nitric acid (trace metal grade) for the determination of dissolved P, K, Ca, Mg, Al, Fe, Mn, and Na concentrations using inductively coupled plasma atomic emission spectrometer (ICP‐AES; Profile Plus, Teledyne/Leeman Labs, Hudson, NH). Blanks, blank spikes, and matrix spikes were included for the quality assurance and control for the ICP‐AES analysis (USEPA, [Link]). As reported in the literature (Pontieri et al. 2014), all four seed samples were enriched with Mg and Fe (Table S2).

Acid‐butanol assay utilizes oxidative cleavage of proanthocyanidins at 4,8 linkages to form red anthocyanidin pigment having *λ*_max_ of 550 nm (Porter et al. 1985). Briefly, 6 mL of n‐butanol + conc. HCl (95:5 vol%) and 1 mL acetone‐water extract were added to a culture tube. After adding 0.2 mL of 2% (w/v) NH_4_Fe^III^(SO_4_)_2_ (in 2 mol L^−1^ HCl), the reactor was vortexed and then placed in boiling water bath for 50 min. Full spectrum (280–650 nm) was taken before and after boiling, and absorbance was recorded at 550 nm. Blank spectra were obtained for each extract before boiling. Calibration was obtained by repeating above‐described procedures for 1–5 mg L^−1^ delphinidin chloride.

Vanillin assay for meta‐substituted flavanoids was conducted following the literature protocol (Price et al. 1978). Briefly, 1 mL of methanol extracts (20 g L^−1^seed in methanol) was reacted with 5 mL of working reagent (2.5 mL of 1% vanillin + 2.5 mL of 8% HCl) in 30°C water bath for 20 min, and the absorbance was recorded at 500 nm. Above‐described procedure was repeated to obtain (1) blanks (1 mL of sample and 5 mL of 4% HCl) and (2) five‐point calibration (1 mL of catechin standards + 5 mL of working reagent).

The DPPH (2,2‐diphenyl‐1‐picrylhydrazyl) is a persistent free radical stabilized by *π* conjugation and sterically (Tang et al. 2006), and are quenched by hydroquinone as well as (poly)phenolic extracts of sorghum seeds (Awika et al. 2003). The DPPH radical quenching assay followed the literature protocol (Berger et al. 2008). Briefly, 4 mL of DPPH dissolved in methanol (0.01 g L^−1^) was added to 4 mL of methanol extracts (20 g L^−1^seed in methanol) at successive dilutions to achieve 20–60% inhibition. Five‐point calibration was obtained using trolox to determine trolox equivalent (in *μ*g trolox g^−1^ seed). One‐way ANOVA of DPPH results were performed using MATLAB version 8.5.0.197613 (R2015a) (Mathworks, Natick, MA) with PLS toolbox version 8.0.1 (Eignevector Research, Manson, WA).

### Fluorescence EEM spectrophotometry with PARAFAC

Fluorescence excitation‐emission (EEM) spectra of methanol and acetone‐water extracts for each seed sample were obtained without dilution using F‐7000 spectrofluorometer (Hitachi, San Jose, CA) set to 220–500 nm excitation and 280–730 nm emission wavelengths in 5 nm intervals; 5 nm excitation and emission slits; 0.5 sec response time; and 2400 nm per min scan speed. As described in detail elsewhere (Stedmon and Bro 2008), parallel factor analysis (PARAFAC) models three‐way data (samples, excitation wavelengths, and emission wavelengths) by minimizing the sum of squares of the residuals. The blank EEM for background solution (methanol and acetone‐water) was obtained daily, and was subtracted from each sample to remove the lower intensity Raman scattering (Christensen et al. 2005). After the removal of additional regions dominated by Rayleigh and Raman peaks and the region without fluorescence, PARAFAC modeling (Stedmon and Bro 2008) was conducted with non‐negativity constraint using MATLAB version 8.5.0.197613 (R2015a) with PLS toolbox version 8.0.1. For methanol extracts, raw EEM spectra were further preprocessed by normalization to the maximum intensity of each seed sample. On the basis of (1) residual/leverage analysis, (2) comparison with the raw EEM spectra, and (3) core consistency diagnostic scores of 2–7 component models, 3 component model was selected to interpret PARAFAC results of methanol and acetone‐water extracts.

## Results and Discussion

### Characterization of seed samples by DPPH, acid‐butanol, ferrozine, vanillin, and UV/vis spectra

Table 1 presents the characteristics of 14 U.S. grain sorghum hybrids, four sweet sorghum, and four African grain sorghum seeds: DPPH radical quenching (in *μ*g trolox g^−1^ seed), acid‐butanol assay (in *μ*g delphinidin g^−1^ seed), reduction of 50 *μ*mol L^−1^ Fe(III), vanillin assay (in mg catehin g^−1^ seed), *λ*_max_, and absorbance at 210 nm. All values are given as mean ± S.D. of triplicate seed extractions. For the U.S. grain sorghum seeds, aphid/webworm/bird resistance responses information was available in the literature, and are provided as fair (F), good (G), and very good (VG) (Ni et al. 2014).

**Table 1:** Characteristics of U.S. grain sorghum hybrids (aphid, webworm, and bird resistance are given as fair (F), good (G), and very good (VG)), sweet sorghum, and African grain sorghum seeds: DPPH radical quenching (in *μ*g trolox g^−1^ seed), acid‐butanol assay (in *μ*g delphinidin g^−1^ seed), reduction of 50 *μ*mol L^−1^ Fe(III), vanillin assay (in mg catehin g^−1^ seed), *λ*_max_, and absorbance at 210 nm

Seed category	Seed label	Aphid	Web	Bird	DPPH (*μ*g trolox g^−1^ seed)	Acid‐butanol (*μ*g delphinidin g^−1^ seed)	FerrozineFe^II^ (*μ*mol L^−1^)	Vanillin (mg cateching^−1^ seed)	*λ*_max1_(nm)	*λ*_max2_(nm)	A(210 nm)
		Worm									
U.S. grain sorghum	4401	F	G	VG	0.54 ± 0.05	2.53 ± 1.70	b.d.l.	b.d.l.	210	N/A	0.31 ± 0.02
	4409	G	G	G	0.67 ± 0.01	b.d.l.	b.d.l.	b.d.l.	210	N/A	0.35 ± 0.02
	4402	F	F	G	0.80 ± 0.03	b.d.l.	b.d.l.	b.d.l.	210	N/A	0.41 ± 0.11
	4422	F	F	VG	0.75 ± 0.06	b.d.l.	b.d.l.	b.d.l.	210	N/A	0.58 ± 0.45
	4411	G	F	VG	0.46 ± 0.13	b.d.l.	b.d.l.	b.d.l.	210	N/A	0.27 ± 0.03
	4434	F	F	G	0.35 ± 0.07	b.d.l.	b.d.l.	b.d.l.	210	N/A	1.03 ± 0.34
	4433	G	G	G	0.62 ± 0.04	b.d.l.	b.d.l.	b.d.l.	210	N/A	0.92 ± 0.37
	4426	F	F	VG	0.20 ± 0.07	b.d.l.	b.d.l.	b.d.l.	210	N/A	0.85 ± 0.06
	4425	G	G	VG	0.27 ± 0.14	b.d.l.	b.d.l.	b.d.l.	210	N/A	0.30 ± 0.11
	4419	G	F	VG	0.61 ± 0.13	b.d.l.	b.d.l.	b.d.l.	210	N/A	0.52 ± 0.18
	4432	F	G	VG	0.59 ± 0.03	b.d.l.	b.d.l.	b.d.l.	210	N/A	0.70 ± 0.20
	4435	G	F	G	0.61 ± 0.19	b.d.l.	b.d.l.	b.d.l.	210	N/A	0.75 ± 0.42
	4437	F	F	G	0.25 ± 0.01	b.d.l.	b.d.l.	b.d.l.	210	N/A	0.51 ± 0.04
	4436	F	F	VG	0.49 ± 0.15	b.d.l.	b.d.l.	b.d.l.	210	N/A	0.97 ± 0.43
sweet sorghum	dale				0.26 ± 0.06	55 ± 1	b.d.l.	2.4 ± 0.2	232	N/A	b.d.l.
	theis				0.81 ± 0.05	60 ± 1	b.d.l.	0.7 ± 0.9	232	N/A	b.d.l.
	keller				9.29 ± 1.50	3672 ± 198	26.5	26.5 ± 0.1	226	282	0.92 ± 0.02
	M81E				28.48 ± 1.52	8892 ± 44	31.1 ± 1.0	94.0 ± 4.6	232	282	0.98 ± 0.02
Africa grain sorghum	seed1				6.04 ± 1.49	2269 ± 86	33.2 ± 0.2	30 ± 5	224	282	1.68 ± 0.00
	seed2				0.11 ± 0.10	72 ± 68	7.2 ± 0.5	b.d.l.	210	N/A	0.63 ± 0.27
	seed3				0.14 ± 0.03	b.d.l.	7.7 ± 1.0	b.d.l.	210	N/A	0.79 ± 0.22
	seed4				18.43 ± 1.11	2909 ± 33	34.8 ± 1.7	61 ± 1	230	282	1.64 ± 0.01

Values are given as mean ± S.D. for triplicate seed extracts. Values below detection limit are denoted b.d.l. N/A indicates that *λ*_max2_ does not exist.

For the U.S. grain sorghum seed extracts, the values were below detection limit for acid‐butanol, ferrozine, and vanillin assays. In addition, a single *λ*_max_ was observed at low wavelength of 210 nm. These observations indicate low chromophore contents of the commercial U.S. grain sorghum hybrids. The U.S. grain forage sorghums are intentionally made low‐tannin to maximize the digestion when fed to livestock (Price and Butler 1977). In Table 1, no clear trend was observable between the DPPH radical quenching (in *μ*g trolox g^−1^ seed) and the resistance to aphid/webworm/bird (Ni et al. 2014). One‐way ANOVA of DPPH results for the U.S. grain sorghum seeds in Table 1 indicated significant difference among hybrids (*P* = 8.98 × 10^−8^). Multicomponent one‐way ANOVA (Table S3) indicated significant (*P* ≤ 0.05) difference in DPPH values between the seed 4437 and eight other seeds (of 14 total hybrids), 4426 with five other seeds, and 4425 with four other seeds; seeds 4409, 4402, 4422, and 4433 were significantly different from seeds 4437, 4426, and 4425. For absorbance at 210 nm (Table 1), one‐way ANOVA for triplicate methanol extracts indicated significant difference (*P* = 0.005) among 14 U.S. grain sorghum seeds; however, multicomponent one‐way ANOVA did not show significant (*P* ≤ 0.05) difference, because of the large error in the absorbance unit. Two‐way ANOVA on the trolox and A(210 nm) analyses showed a significant difference between seeds (*P* = 0.0005) as well as the analyses (trolox and A(210 nm), *P* = 0.0381), and indicated interactions between the seeds and analysis method (*P* = 0).

In contrast to the U.S. grain sorghum, values were above detection limit for the following tannin‐rich (based on the acid‐butanol assay in Table 1) sweet sorghum and African grain sorghum seeds for all analytical methods: keller, M81E, seed 1 and seed 4. Unlike the U.S. grain sorghum, there were two *λ*_max_ at 230 and 282 nm in these samples (seed 1, seed 4, M81E and Keller, see Fig. S3 for the full spectra). In contrast, Dale and Theis showed low absorbance with one *λ*_max_ near 210 nm, much like the U.S. grain sorghum (Table 1). Linear correlations were observed between trolox and delphinidin (radical quenching vs. proanthocyanidins; *r* ^*2* ^= 0.99 for sweet sorghum and *r* ^*2* ^= 0.81 for African grain sorghum, Fig. S2) as well as trolox and catechin (radical quenching vs. flavonoids; *r* ^*2* ^= 0.99 for 4 sweet sorghum varieties and *r* ^*2* ^= 0.97 for 4 African grain sorghum varieties, Fig. S2).

### EEM‐PARAFAC of acetone‐water and methanol extracts

**Figure 1:**
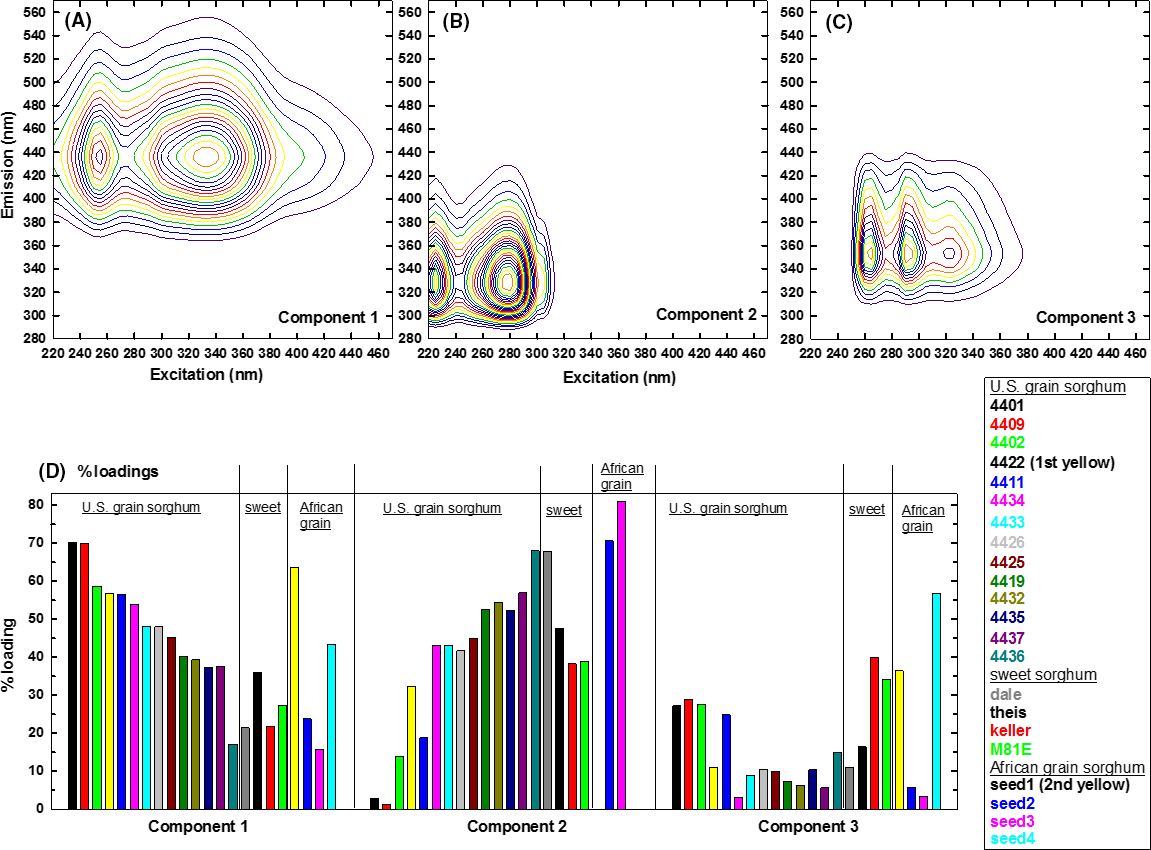
Three component (excitation‐emission) EEM/PARAFAC (parallel factor analysis) fingerprints (A–C) and % contribution (D) of methanol extracts. Raw EEM spectra were normalized to the maximum intensity prior to PARAFAC.

Figure 1A–C present 3 component EEM fingerprints obtained by PARAFAC analyses of methanol extracts. The total of 22 raw EEM spectra was normalized to the maximum intensity such that each sample will have equal impact on the PARAFAC model. This preprocessing procedure enabled us to focus on the variations among seeds, rather than the absolute magnitude of EEM intensity. Selected raw spectra of methanol extracts are provided in Figure S4. Component 2 (lowest Ex/Em wavelengths) of methanol extracts was the primary contributor to seed 2 and seed 3. A longer emission wavelength indicates more conjugated, aromatic, condensed, and higher MW structures (Lichtman and Conchello 2005). Component 1 had higher Ex/Em wavelengths than Component 2, and showed an opposite contribution trend of Component 2. For the U.S. grain sorghum, the contribution of Component 1 decreased from the left (4401) to right (4436), while the opposite trend was observed for Component 2 (Figure 1D). Overall, Component 2 (lowest Ex/Em) was characterized by high contribution to seed 2, seed 3, and Dale. Component 1 behaved oppositely to Component 2: high contributions to seed 1 and seed 4, and decreasing contribution to U.S. grain sorghum from left (4401) to right (4436). Component 3 strongly contributed to seed 1, seed 4, Keller, and M81E.

**Figure 2:**
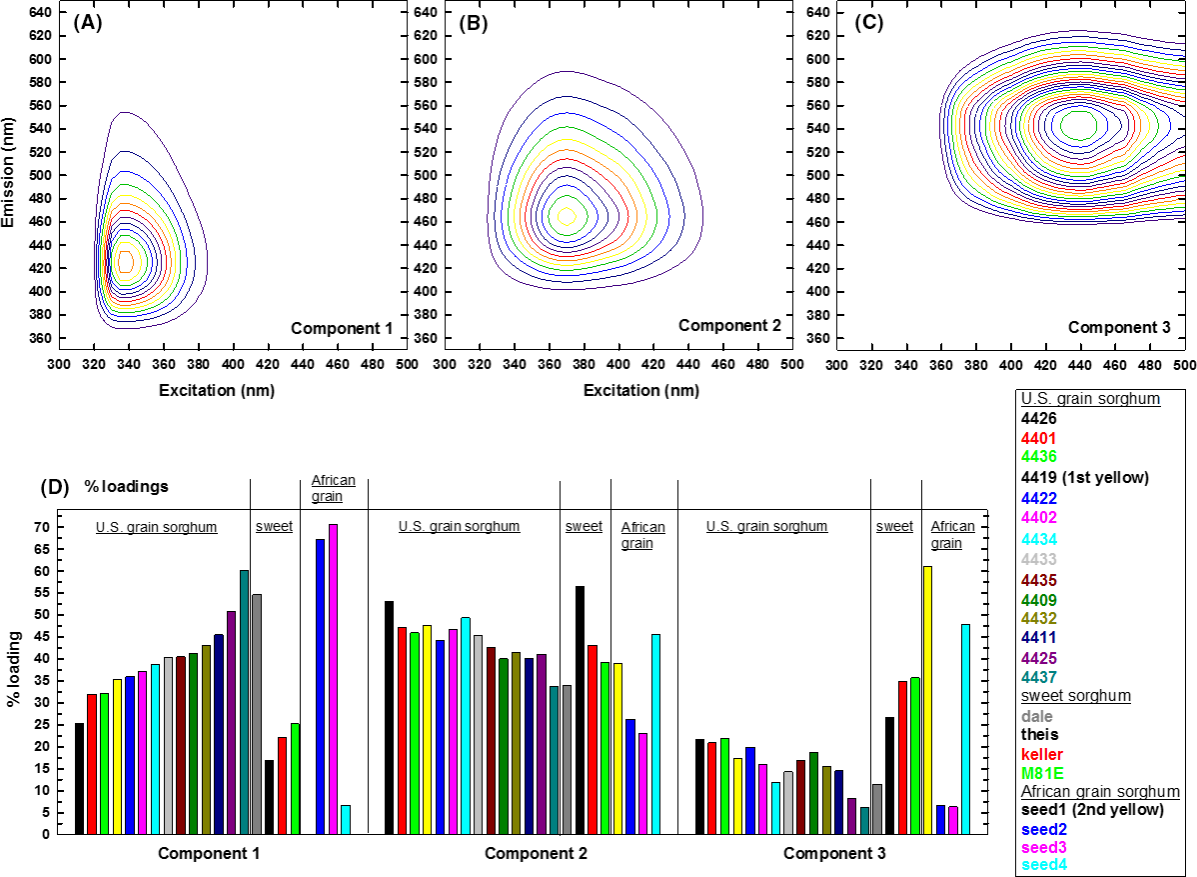
Three component (excitation‐emission) EEM/PARAFAC (parallel factor analysis) fingerprints (A–C) and % contribution (D) of acetone/water (70/30 v/v) extracts.

For acetone‐water extracts, Component 1 showed the lowest Ex/Em wavelengths (Fig. 2A), and was the primary contributor to seed 2 and seed 3, and Dale (Fig. 2D), similarly to the Component 2 (lowest Ex/Em wavelengths) of methanol extracts. Oppositely, Component 3 has the highest Ex/Em wavelengths (Fig. 2C), and was the primary contributor to seeds 1 and 4 (Fig. 2D). In conclusion, low Ex/Em fingerprints (Component 2 of methanol and Component 1 of acetone‐water) were the primary contributor to African (seed 2 and seed 3) and sweet (Dale) sorghum having low DPPH, acid‐butanol, ferrozine, and vanillin responses in Table 1. Oppositely, high Ex/Em fingerprints (Component 1 of methanol and Component 3 of acetone‐water) were the primary contributors to seed 1 and 4.

To visualize the relationships, linear correlation was obtained between DPPH radical quenching (Table 1 in in *μ*g trolox g^−1^ seed) and % PARAFAC contributions (Figs. 1, 2). Component 3 of methanol extract (Fig. 1) correlated with DPPH radical quenching (*r* ^*2* ^= 0.91, Fig. S5); linear correlation was observed for sweet sorghum seeds when M81E was removed (*r* ^*2* ^= 0.98). No correlation was observed for the U.S. grain sorghum. For acetone/water, Component 2 correlated with trolox of seeds 1–4, and there was no correlation for sweet sorghum or U.S. grain sorghum. In conclusion, DPPH radical quenching correlated with flavonoids and proanthocyanidin contents of African grain and sweet sorghum seeds (Fig. S2); no correlation was observable for the U.S. grain sorghum hybrids. These characteristics of African grain, sweet, and U.S. grain sorghum seeds correlated with the degree of conjugation (Lichtman and Conchello 2005) of EEM/PARAFAC fingerprints (Figs 1, 2).

## Conflict of Interest

None declared.

## Supporting information


**Data S1.** Physical properties of seed samples.
**Data S2**. E_h_‐pH diagram of phenolic compounds.
**Data S3.** Metal contents of selected seed samples.
**Data S4.** Multicomponent one‐way ANOVA results for Table 1.
**Data S5.** Linear relationships between radical quenching and acid‐butanol/vanillin (Table 1).
**Data S6.** Raw EEM spectra of selected seed extracts.
**Data S7.** Linear relationships between radical quenching and acid‐butanol/vanillin assays in Table 1.
