# Supplementary material for: Structure‐reactivity relationships between the fluorescent chromophores and antioxidant activity of grain and sweet sorghum seeds
Source: Food Sci Nutr. 2016 Feb 16;4(6):811–7. doi: 10.1002/fsn3.350 (PMC5090644; doi:10.1002/fsn3.350)
Supplement: Supplementary file 1 — Data S1. Physical properties of seed samples. Data S2. Eh‐pH diagram of phenolic compounds. Data S3. Metal contents of selected seed samples. Data S4. Multicomponent one‐way ANOVA results for Table 1. Data S5. Linear relationships between radical quenching and acid‐butanol/vanillin (Table 1). Data S6. Raw EEM spectra of selected seed extracts. Data S7. Linear relationships between radical quenching and acid‐butanol/vanillin assays in Table 1. [file FSN3-4-811-s001.doc]

Supporting Information for

Structure-Reactivity Relationships between the Fluorescent Chromophores and Antioxidant Activity of Grain and Sweet Sorghum Seeds

Minori Uchimiya^*,a^, Xinzhi Ni^b^, Ming Li Wang^c^

*^a^USDA-ARS Southern Regional Research Center, 1100 Robert E. Lee Boulevard, New Orleans, Louisiana 70124*

*^b^USDA-ARS Crop Genetics and Breeding Research Unit, 2747 Davis Road, Tifton, Georgia 31793*

*^b^USDA-ARS Plant Genetic Resources Conservation Unit, Griffin, Georgia 30223*

*Corresponding author fax: (504) 286-4367, phone: (504) 286-4356, email: sophie.uchimiya@ars.usda.gov

Number of pages: 6

Number of tables: 3

Number of figures: 5

**I. Physical properties of seed samples**

**Table S1.** Condensed tannins-rich seeds: radical quenching µ flavonoids µ e^-^ donating capacity

**II. E_h_-pH diagram of phenolic compounds**


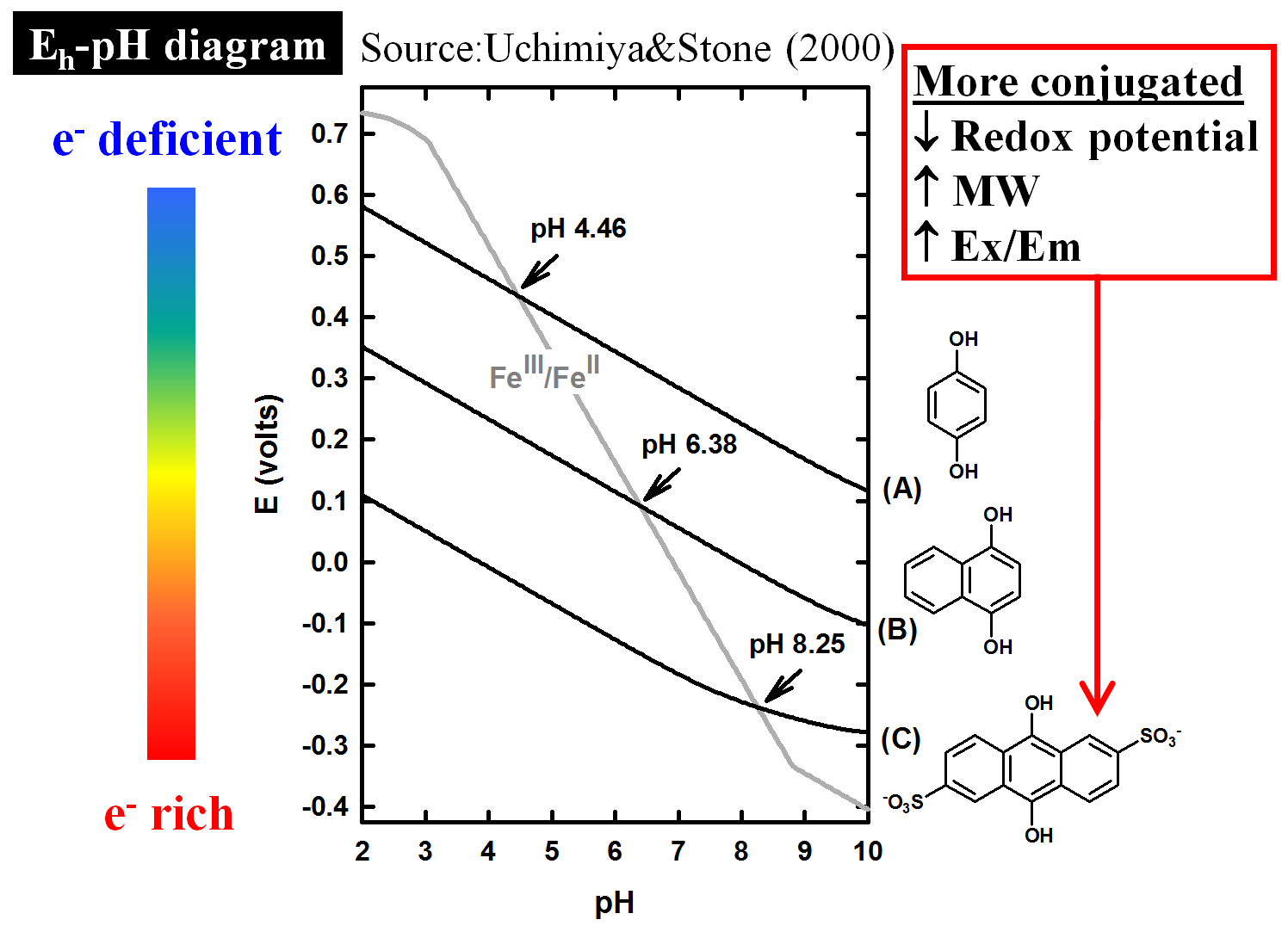


**Figure S1.** E_h_-pH diagram of model phenolic compounds.

**III. Metal contents of selected seed samples**

**Table S2.** Total K, P (in mg g^-1^ seed), Mg, Fe, Na, Ca, Mn, and Zn (in μg g^-1^ seed) contents of selected seed samples extracted using ammonium oxalate

**IV. Multi-component one-way ANOVA results for Table 1**

**Table S3.** DPPH radical quenching (in μg trolox g^-1^ seed, Table 1) values that are significantly different at P≤0.05 (*), 0.01 (**), and 0.001 (***) levels of probability (one-way ANOVA for triplicate seed extracts)

**V. Linear relationships between radical quenching and acid-butanol/vanillin (Table 1)**

**
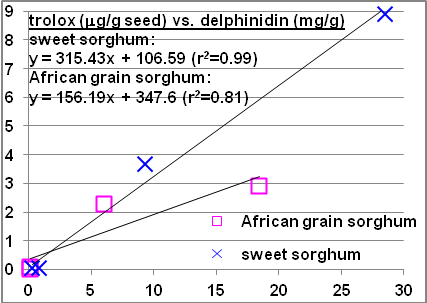
**

**
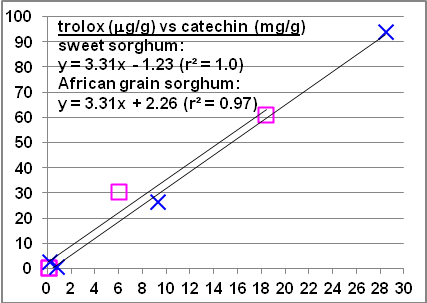
**

**Figure S2.** Linear relationships observed in Table 1 for sweet sorghum and African grain sorghum seeds.

**
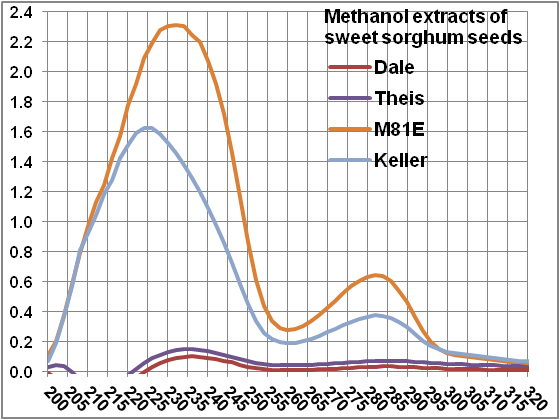
**

**Figure S3.** Sweet (M81E&Kellter) and African grain sorghum seeds (1&4) containing high condensed tannins (2-9 mg delphinidin equiv./g seed) had two λ_max_ at 230 and 280 nm; only one λ_max_ (210 nm) in low-tannin U.S. and African grain sorghum seeds.

**VI. Raw EEM spectra of selected seed extracts**

**
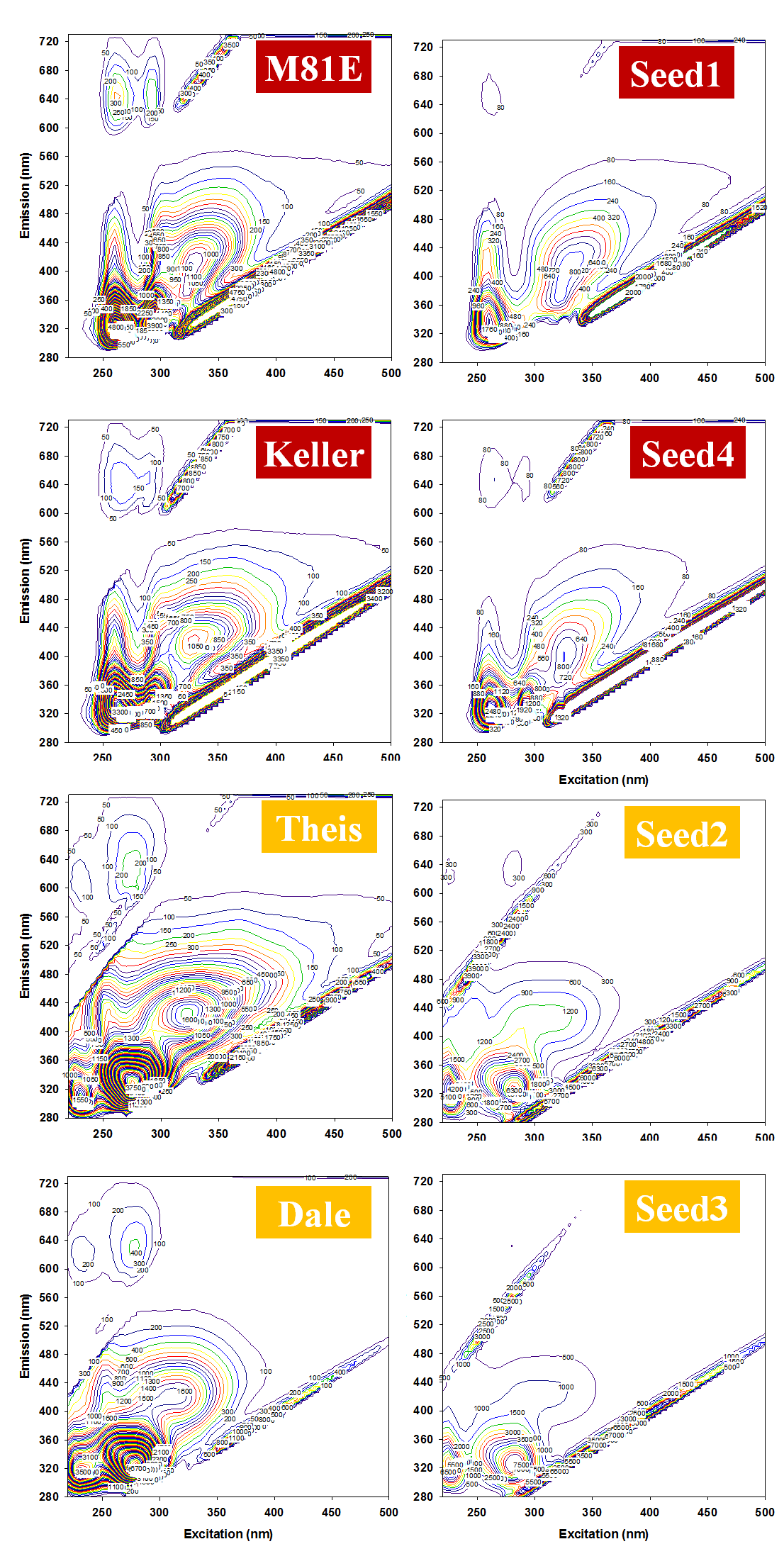
**

**Figure S4.** Raw EEM spectra of methanol extracts.

**VII. Linear relationships between radical quenching and acid-butanol/vanillin assays in Table 1**

**
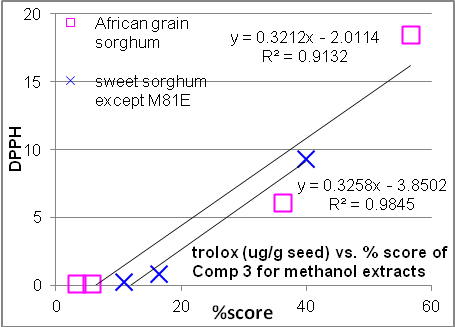
**

**
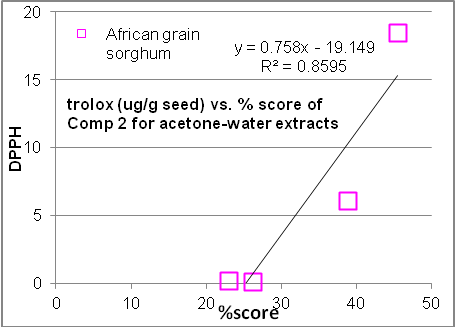
**

**Figure S5.** Linear relationships observed in between PARAFAC fingerprints and DPPH radical quenching for sweet sorghum and African grain sorghum seeds.
